# Stakeholder perceptions of gaps in antimicrobial resistance mitigation: a multinational multisectoral survey to inform prioritization of One Health interventions

**DOI:** 10.1017/ash.2026.10750

**Published:** 2026-06-10

**Authors:** Godwin Pius Ohemu, Howard Rodriguez-Mori, Marcelo Schmidt, Babafela Awosile

**Affiliations:** School of Veterinary Medicine, Texas Tech University, Amarillo, USA

## Abstract

**Objective:**

This study examined stakeholder perceptions of gaps in antimicrobial resistance (AMR) mitigation efforts across different sectors and career stages, with the goal of generating insights to support prioritization of One Health interventions.

**Method:**

We conducted a cross-sectional survey of AMR stakeholders using a structured questionnaire between October and November 2025. Stakeholders were recruited through international networks across 33 countries on 5 continents. Respondents listed the top three areas in AMR mitigation efforts that they perceived as receiving insufficient attention but require greater focus to reduce the emergence and spread of drug-resistant bacterial infections. We performed thematic analysis, calculated weighted priority scores, conducted an Impact-Effort Matrix analysis, and applied Fisher’s exact tests to examine associations between themes, professional sector and career stages.

**Results:**

Among 103 respondents, participants spanned nine professional sectors and four career stages. Antimicrobial stewardship (18.8%, weighted score 119) and Environmental AMR and Waste (16.5%, weighted score 108) were the most frequently cited perceived gaps. Impact-Effort Matrix analysis identified four priority areas as Quick Wins (stewardship, IPC, surveillance, and animal health, accounting for 45.7% of impact), two Major Projects (environmental AMR, R&D), three Fill-ins (awareness, One Health, other/cross-cutting), and three Hard Slogs (access, financing, policy). No significant associations were observed between perceived mitigation gaps across professional sector and career stages.

**Conclusions:**

The emphasis on antimicrobial stewardship among stakeholders highlights a perceived disconnect between stewardship policy adoption and on-the-ground practice. The Impact-Effort Matrix provides a decision-support tool that may assist policymakers and funders in prioritizing One Health interventions.

## Introduction

The phenomenon whereby disease-causing organisms become resistant to the pharmaceutical agents designed to kill or inhibit their growth is a critical global health concern. This phenomenon, known as antimicrobial resistance (AMR), resulted in 1.27 million deaths in 2019 and is estimated to cause 10 million deaths annually by 2050 if effective intervention strategies are not implemented.^1,2^ The United States, for instance, records 2.8 million antibiotic-resistant infections every year, with more than 35,000 deaths.^3^

While numerous studies have documented the epidemiology of AMR and various policy interventions,^4–6^ few have systematically captured stakeholders ’ perceptions of gaps in current AMR mitigation efforts. Understanding these gaps can inform strategic resource allocation and identify blind spots in intervention approaches.^7^

To address this gap, we conducted a cross-sectional survey of AMR stakeholders using a structured questionnaire to identify areas in AMR mitigation efforts that stakeholders perceive to be receiving insufficient attention but requires greater focus to reduce the emergence and spread of drug-resistant bacterial infections. Rather than asking respondents to rank predetermined perceptions, we used an open-ended questionnaire to capture emerging perceptions across sectors and career stages.

This study aims to examine stakeholder perceptions of gaps in AMR mitigation efforts across professional sectors and career stages, and to apply an Impact-Effort Matrix to support prioritization of One Health interventions. The insights from this study can help policymakers, funders, and implementers optimize resource allocation to address the most critical but yet under-addressed aspects of AMR mitigation within the One Health domain.

## Methodology

### Study design and participants

We conducted a cross-sectional survey to identify stakeholders’ perceptions of gaps in AMR mitigation efforts across the One Health domain. For this study, we defined stakeholders as individuals with active professional engagement in AMR related work, including research, clinical practice, laboratory diagnostics, public health program management, policy development, regulatory oversights, or advocacy. This definition reflects our study’s focus on those whose professional roles position them to observe, respond to or influence AMR mitigation efforts across human, animal and environmental interface. Participants were recruited through professional AMR networks across 33 countries on 5 continents via mailing lists, direct email, and LinkedIn posts. The survey was administered in English using convenience sampling. No formal sample size calculation was performed. Of the 108 individuals who accessed the survey, five were excluded: two declined consents, one did not specify their duration of experience in AMR, one indicated not working in AMR, and one entered “None” for years of AMR experience. The final sample comprised 103 stakeholders who contributed 309 priority statements. The response rate of this survey could not be calculated because the survey was distributed through multiple open channels.

### Survey instrument

The questionnaire for this study consisted of a single open-ended question asking respondents to identify their top three under addressed AMR priorities. Additional metadata collected included the country of work (free-text), continent of work (self-selected by respondents from a predefined list of six options: Africa, Asia, Europe, North America, South America, and Oceania), primary sector of work (self-selected from nine predefined categories: Research and Development, Research/Academic, Clinical, Laboratory, Policy, Program Management, NGO, Government Agency, and Other), and years of experience in AMR (free-text). No country-to-continent assignments or reclassifications were made by the research team. Although respondents reported their country of work and continent, geographic comparisons were not performed due to unequal continent representation, with North America contributing ten respondents and South America one. Responses were coded as Top 1, Top 2, and Top 3 with corresponding weights: Top 1 = 3, Top 2 = 2, Top 3 = 1.

### Data cleaning and thematic analysis

Data was cleaned in Microsoft Office Excel 2016 and restructured into long format yielding 309 priority statements. A thematic codebook was developed using inductive coding and deductive alignment with One Health priority domains outlined by the Quadripartite Joint Secretariat (WHO, FAO, WOAH, UNEP)^4,6^ and published AMR priority-setting studies.^8–11^ The final codebook consisted of 12 thematic categories with definitions, inclusion and exclusion criteria, and example statements, applied consistently across all responses. We acknowledge that boundary areas exist between some themes; for example, Environmental AMR and Waste and One Health Integration and Coordination share conceptual boundaries around cross-sector coordination. The codebook inclusion and exclusion criteria were explicitly designed to minimize such overlap, and any disagreements in boundary cases were resolved through discussion and consensus between the two coders, with refinements made to the codebook definition as needed. All responses were coded by the first author using the finalized codebook, while the last author independently reviewed all coded responses. Disagreements regarding theme assignment were discussed between the two authors and resolved through consensus, with refinements made to the codebook definitions as needed to improve clarity.

### Data analysis and visualization

Data were analyzed in R version 4.4.2 (R Core Team 2024). The overall analytical sequence was as follows: (1) thematic coding of all 309 priority statements using the finalized codebook; (2) weighted scoring of each theme based on ranking position; (3) statistical testing of associations between themes and respondent characteristics; and (4) Impact-Effort Matrix classification to support strategic prioritization. Illustrative quotes were selected from the coded responses to represent the range of perspectives within each theme, prioritizing statements that clearly expressed the theme or added context beyond the quantitative findings alone. Weighted scores were computed, and associations were tested using Fisher’s exact tests (*P*<.05). Themes were mapped onto an Impact-Effort Matrix as demonstrated in recent studies.^12,13^ Effort Scores were assigned through structured author consensus on a 5-point scale (1 = very easy to implement, 5 = very difficult to implement). The effort score assessment considered four dimensions: (1) resource requirements, (2) implementation complexity, (3) regulatory and political feasibility, and (4) time frame to achieve measurable impact. These ratings were informed by AMR implementation studies.^14–16^

### Ethics and clearance

This study was exempted from full ethics committee review and approval because it only collected stakeholders’ opinion on perceived gaps in AMR mitigation efforts, and no personal identifiable information was collected or retained.

## Result

A total of 103 stakeholders from nine sectors across four career stages participated in the study (Table 1), with a median of 7 years of experience in AMR. Respondents represented 33 countries across five continents, with the highest participation from Nigeria (n = 17) and Kenya (n = 14), followed by South Africa (n = 8), the United Kingdom (n = 8), and India (n = 6). Six respondents reported working at a global level and were not assigned to a specific country. Country of work was provided as a free-text response. Continent was self-selected from a predefined list of six options (Africa, Asia, Europe, North America, South America, and Oceania); no assignments or reclassifications were made by the research team. A full geographic distribution is provided in Supplementary file S3. A standardized thematic codebook was developed to classify AMR priority statements (Table 2). Across 309 priority statements, antimicrobial stewardship and use ranked highest while environmental AMR and waste was the most cited Top 1 priority theme (Table 3). Infection prevention and control (IPC/WASH) increased after weighting, whereas One Health integration and policy-focused themes were least prioritized by stakeholders. To illustrate the character of responses within leading themes, selected quotes are presented below:

**Table 1.** Professional sector and experience level distribution of stakeholders included in the study (n = 103) Table 1 long description.

Characteristic	Category	n	%	(95% CI)
Total respondents		103		
Sector	Research/Academic	44	42.7	33.2–52.6%
Sector	Clinical	20	19.4	12.4–28.4%
Sector	Laboratory	11	10.7	5.6–18.2%
Sector	Research & Development	8	7.8	3.5–14.7%
Sector	Other	7	6.8	2.9–13.5%
Sector	NGO	6	5.8	2.3–12.3%
Sector	Government Agency	5	4.9	1.7–11.0%
*Sector	Program Management	1	1.0	0.0–5.3%
*Sector	Policy	1	1.0	0.0–5.3%
Years working in AMR	0–4 years	33	32.0	3.4–41.7%
Years working in AMR	5–9 years	26	25.2	17.4–34.6%
Years working in AMR	10–19 years	25	24.3	16.6–33.7%
Years working in AMR	>20 years	19	18.4	11.6–27.2%
Years working in AMR (continuous)		Median 7.0 (IQR 3.0–15.0)		5.0–9.0

Values are presented as counts and percentages. Years of AMR experience are summarized as median and interquartile range (IQR). Percentages may not total 100 due to rounding. *n = 1 categories were included for completeness but not interpreted statistically.

**Table 2.** Theme code book for classification of stakeholders perceived gaps in AMR mitigation efforts with exclusion, inclusion, and examples Table 2 long description.

Theme	Definition	Inclusions	Exclusions	Example
**Antimicrobial stewardship and use**	Issues related to inappropriate, excessive, or irrational use of antimicrobials across human, animal, or community settings.	Overuse, misuse, OTC sales, irrational prescribing, and lack of stewardship programs.	Veterinary or farm-specific AMU issues (Animal Health theme).	“OTC medications and how they contribute to AMR.”
**Environmental AMR and waste**	AMR risks driven by environmental contamination, including wastewater, effluent, soil, water bodies, and pollution.	Wastewater, pharmaceutical waste, soil/water contamination, environmental reservoirs.	Farm biosecurity or animal-related contamination (Animal Health theme).	“Sewage system and wastewater monitoring, since these areas are highly contaminated with antibiotics, non-antibiotics, and other substances.”
**Animal health and food systems**	AMR issues arising from livestock, poultry, aquaculture, veterinary practices, and food production chains.	Livestock AMU, farm biosecurity, veterinary prescribing, foodborne AMR issues.	Environmental contamination unrelated to animal production (Env AMR theme).	“Failure to consider crossover resistance in the same drugs that are used in both humans and animals.”
**Awareness, education and behavior change**	Lack of education, awareness, and behavior change needed to improve antimicrobial use and reduce AMR.	Community sensitization, awareness campaigns, behavior change efforts, knowledge gaps.	Training specific to IPC or laboratory systems.	“Public awareness is the key to solve population related problems.”
**Infection prevention and control (IPC/WASH)**	Deficiencies in hygiene, sanitation, WASH systems, and infection prevention practices increasing infection and antibiotic demand.	Hospital IPC, hand hygiene, WASH, sterilization, biosecurity practices.	Environmental contamination (Env AMR theme).	“Infection prevention and control in hospital settings. By improving healthcare infrastructure.”
**R&D, innovation and diagnostics**	Gaps in research, development of new antibiotics, diagnostics, vaccines, and innovative AMR tools.	New antibiotics, diagnostics, vaccines, rapid testing, AMR R&D investments.	Lab infrastructure for surveillance (Surveillance theme).	“Discovering new agents that are effective against drug-resistant strains.”
**Access, equity and medicine quality**	Inequities in access to quality antimicrobials, including substandard or falsified medicines, and affordability issues.	Substandard/fake antimicrobials, affordability issues, inequitable access.	Misuse or overuse of drugs (Stewardship theme).	“Improve access, but regulate the access to antibiotics.”
**Financing, infrastructure and health systems**	System-level underfunding, inadequate infrastructure, and weak health systems that hinder AMR mitigation.	Funding gaps, workforce shortages, infrastructure weaknesses, systemic resource challenges.	Specific laboratory infrastructure issues (Surveillance theme).	“Lack of motivation by large pharmaceutical companies to develop new front-line antibiotics because the long-term profit margin is low compared to other drug classes.”
**Surveillance, laboratories and data**	Lack of effective AMR/AMU surveillance, monitoring, data quality, laboratory capacity, and reporting systems.	Lab gaps, missing AMR data, monitoring, poor reporting, weak surveillance systems.	Diagnostic innovation (R&D theme).	“Lack of Lab investigation before administering antibiotics.”
**Policy, governance and regulation**	Weak governance, regulation, enforcement, and policy implementation affect AMR control.	Regulation, NAP implementation, enforcement, governance structures.	General multisector coordination issues (One Health Integration).	“Over-the-counter purchase of antibiotics should be banned globally.”
**One health integration and coordination**	Failures in coordination across human, animal, and environmental sectors; siloed AMR efforts.	Multisectoral action gaps, cross-sector collaboration failures, siloed governance.	Specific regulatory or enforcement discussions (Policy theme).	“AST in One Health approach, AMR transmission, Stakeholder integration, AMU in veterinary and human practitioners.”
**Other/cross-cutting**	Broad or mixed statements not fitting into a specific theme or covering multiple unrelated aspects.	Very broad, mixed, or unclear statements without a dominant theme.	Anything clearly fitting into another defined theme.	“Pediatric medicine, Nutrition, Phytotherapy, Tuberculosis”

Themes were derived from One Health AMR global priority domains outlined by the Quadripartite Joint Secretariat (WHO, FAO, WOAH, UNEP). Each theme includes an operational definition, inclusion and exclusion criteria, and illustrative examples to support consistent coding. The codebook was applied to all priority statements to ensure transparency and reproducibility. The “Other/Cross-cutting” category was used sparingly for responses that did not align with a single dominant theme.

**Table 3.** Distribution of perceived gaps/themes by frequency of mention, weighted score, and priority rank across stakeholders’ responses Table 3 long description.

					Priority Rank		
Rank	Perceived gap/Theme	n	%	Weighted Score^†^	Top 1	Top 2	Top 3
**1**	Antimicrobial Stewardship and Use	58	18.8	119	21	19	18
**2**	Environmental AMR and Waste	51	16.5	108	22	13	16
**3**	Infection Prevention and Control (IPC/WASH)	29	9.4	63	9	16	4
**4**	Surveillance, Laboratories and Data	29	9.4	59	10	10	9
**5**	R&D, Innovation and Diagnostics	29	9.4	56	10	7	12
**6**	Animal Health and Food Systems	25	8.1	54	11	7	7
**7**	Awareness, Education and Behaviour Change	26	8.4	52	8	10	8
**8**	Access, Equity and Medicine Quality	24	7.8	41	4	9	11
**9**	Financing, Infrastructure and Health Systems	12	3.9	21	3	3	6
**10**	Other / Cross-cutting	11	3.6	19	2	4	5
**11**	Policy, Governance and Regulation	8	2.6	17	3	3	2
**12**	One Health Integration and Coordination	7	2.3	9	0	2	5

^†^Weighted score calculated as 3 points for Top 1, 2 points for Top 2, and 1 point for Top 3 priorities.

On stewardship, one respondent said: *“Overuse and misuse of antimicrobials by unqualified persons, including quacks, and lack of supervision by trained professionals, especially in LMICs…”* On environmental AMR, another respondent noted: *“I believe that focusing on antibiotic prescriptions is a mistake; we are overlooking a large part of the problem related to the environment and the plastisphere.”* On surveillance, a respondent said: *“Global AMR strategies still focus mainly on hospitals, yet most antibiotic use and transmission occur in the community.”* These perspectives are representative of the broader thematic emphasis observed across the 309 priority statements in the data set.

**Figure 1.**
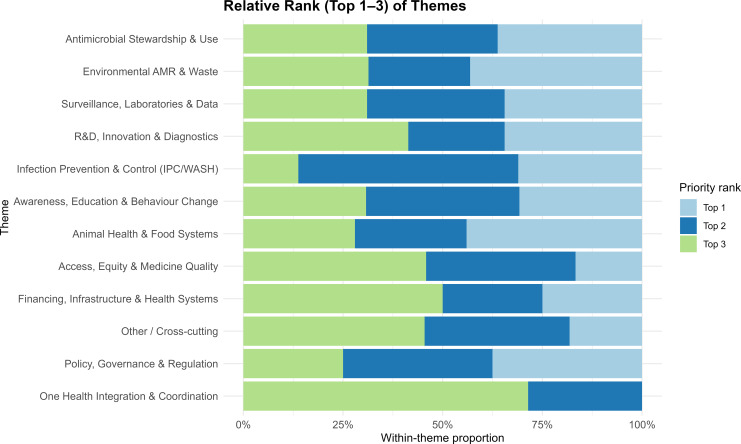
Figure 1 long description. Stacked bar proportional distribution of stakeholders ranked priorities (Top 1 vs 2 vs 3) across perceived gaps/thematic domains, illustrating how relative importance assigned by respondents varies by theme. Bars represent the proportion of mentions assigned to each ranking category within themes. Percentages were calculated using the total number of mentions per theme as the denominator.

**Figure 2.**
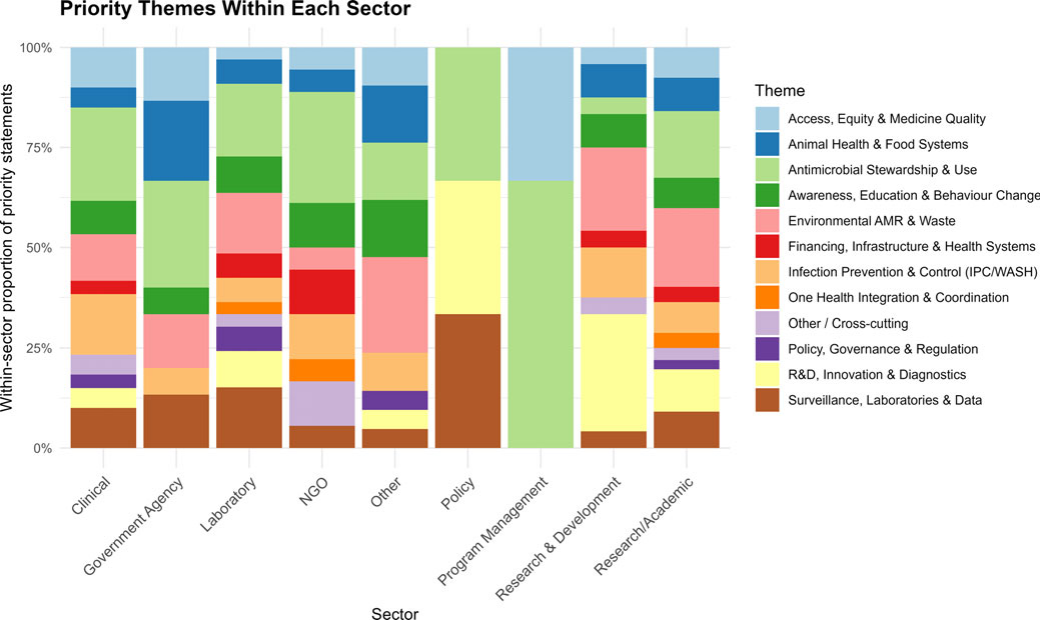
Figure 2 long description. Sector-specific distribution of AMR priority themes showing the proportion of priority statements attributed to each thematic domain within clinical practice, laboratory surveillance, research/academic, and other professional sectors. Sectors with very low representation (n = 1) were included for completeness but were excluded from statistical comparison. Bars represent the proportion of statements assigned to each theme within sectors. No significant associations in perceived AMR mitigation gaps across professional sector were observed. (Fisher’s exact test, *P* = .91).

**Figure 3.**
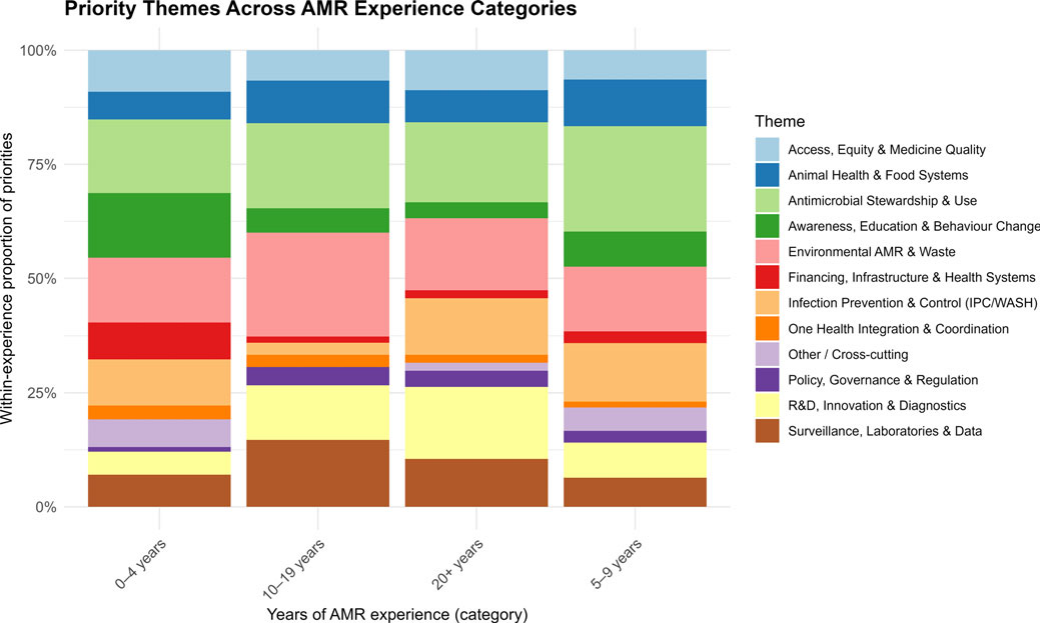
Figure 3 long description. Relative distribution of identified AMR priority themes across respondents stratified by year of professional experience (0–4, 5–9, 10–19, and >20 years), illustrating how perceived gaps vary with level of AMR experience. Figure 3 illustrates the relative distribution of identified AMR priority themes across respondents stratified by year of professional experience. Early career professionals (0–4 years) showed relatively stronger emphasis on Antimicrobial Stewardship and Use (16.2%), Awareness, Education and Behaviour Change (14.1%), and Environmental AMR and Waste (14.1%). Mid-career stakeholders (5–9 years) most frequently prioritized Antimicrobial Stewardship and Use (23.1%), Environmental AMR and Waste (14.1%), and Infection Prevention and Control (IPC/WASH) (12.8%). Respondents with 10–19 years of experience emphasized Environmental AMR and Waste (22.7%), Antimicrobial Stewardship and Use (18.7%), and Surveillance, Laboratories and Data (14.7%). Senior professionals (20+ years) showed preference for Antimicrobial Stewardship and Use (17.5%), Environmental AMR and Waste (15.8%), alongside R&D, Innovation and Diagnostics (15.8%). However, these differences were not statistically significant (Fisher’s exact test, *P* = .13).

Although antimicrobial stewardship and use had the highest overall frequency (n = 58) and weighted priority score (119), environmental AMR and waste received the greatest number of Top 1 priority rankings (n = 22), indicating that while stewardship was most commonly cited across all three ranking positions, respondents most frequently chose environmental AMR as their single most urgent concern followed by animal health and food system (Figure 1). Patterns were similar across sectors and experience levels, with no significant associations (*P* = .91, *P* = .13, Figures 2 and 3). Additional sector and experience specific details are provided in supplementary files S1 and S2.

**Figure 4.**
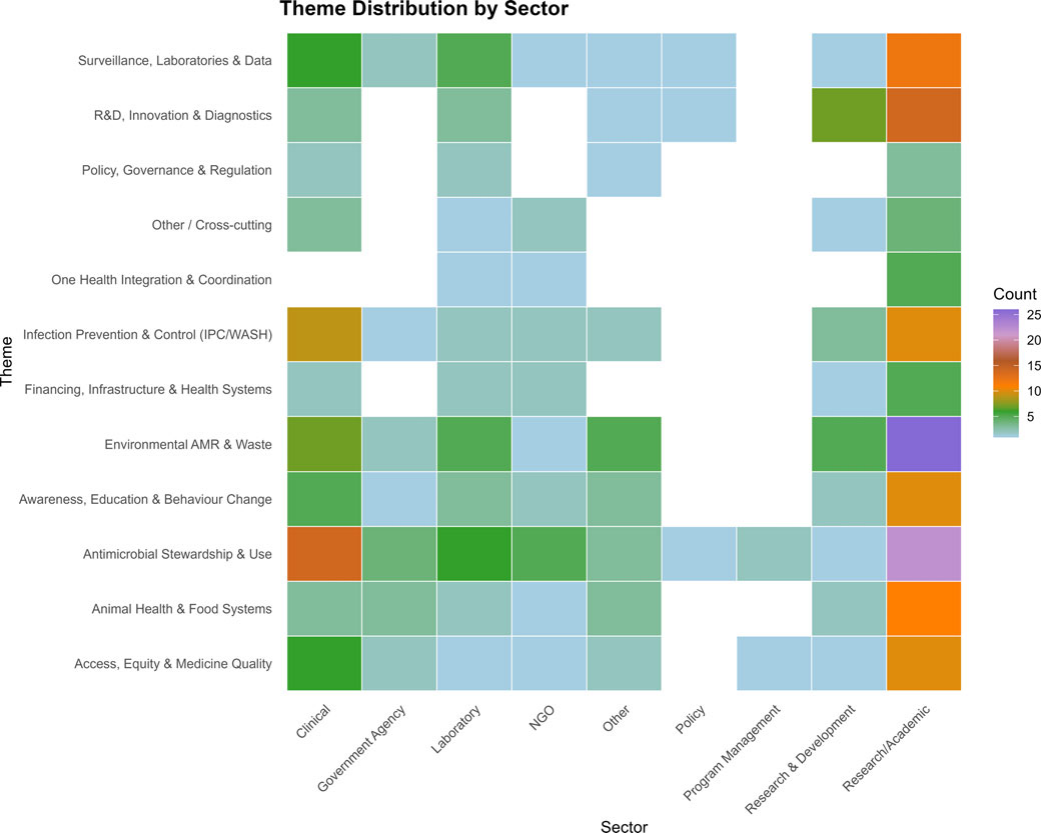
Figure 4 long description. Heatmap illustrating the cross-sectoral distribution and intensity of AMR priority themes across professional sectors, highlighting areas of thematic concentration and relative emphasis within different sectors among stakeholders. Darker shading indicates higher numbers of priority statements within a sector-theme combination.

**Figure 5.**
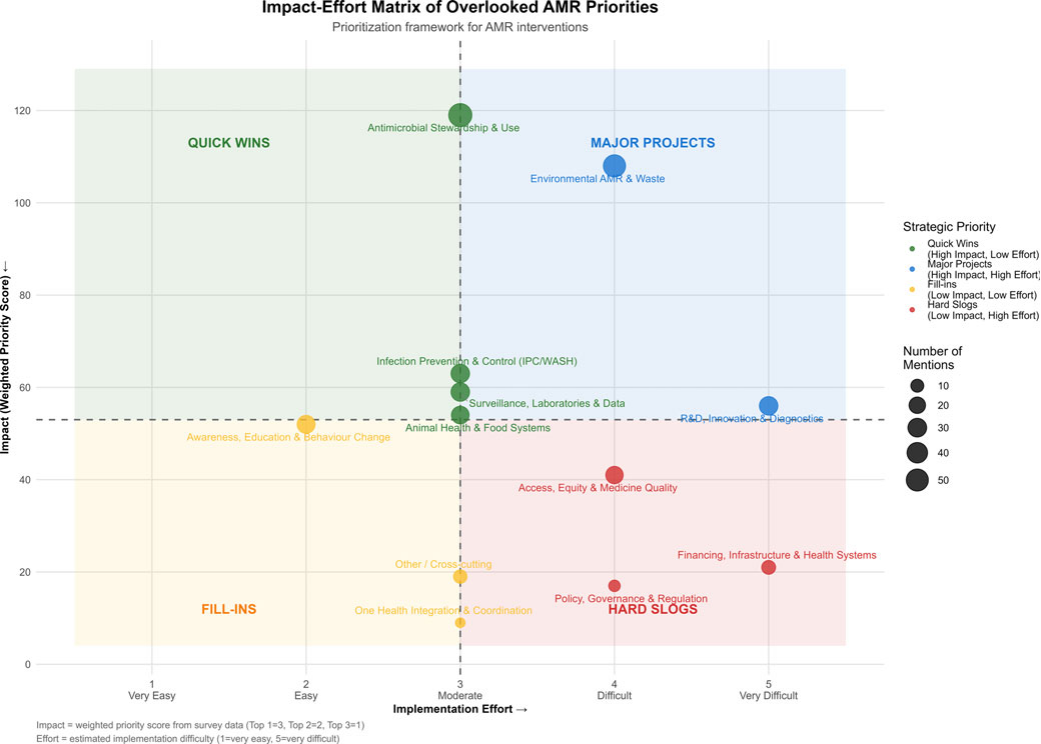
Figure 5 long description. Impact-Effort Matrix of overlooked AMR priority themes identified by stakeholders, illustrating the relative impact (weighted priority score based on Top 1, Top 2, and Top 3 rankings) and implementation efforts (feasibility) to inform strategic prioritization of One Health Interventions. Impact reflects weighted priority scores, and implementation effort was estimated based on resource requirements, infrastructure needs, and regulatory complexity (scale 1-5). Themes are grouped into strategic quadrants (quick wins, major projects, hard slog, and fill-ins).

Research and academic respondents contributed the most priority statements across themes, whereas clinical and laboratory sectors focused on fewer themes (Figure 4). Sector-specific dominant themes and illustrative respondent perspectives are presented in Supplementary file S4. A descriptive breakdown of priority themes by continent of work is provided in Supplementary file S5. Impact-Effort Matrix mapping identified stewardship, IPC, surveillance, and animal health as “quick wins,” while environmental AMR, Innovation, and policy-related themes required greater implementation effort (Figure 5).

## Discussion

This study collected 309 priority statements from 103 AMR stakeholders spanning nine professional sectors across four career stages. Using inductive and deductive thematic coding, we identified 12 AMR priority themes and assessed their prominence across sectors and experience levels working in AMR. This study captures what stakeholders think, observe, or experience regarding areas of AMR mitigation perceived to be insufficiently addressed. These perceptions do not directly quantify the magnitude of existing implementation gaps, rather, they reflect the priorities and concerns of professionals working across the AMR landscape. The findings of this study should therefore be interpreted within this context.

Across analysis, antimicrobial stewardship and use and environmental AMR and waste were consistently cited by stakeholders as insufficiently addressed, accounting for 36.7% of total weighted scores. A second tier included IPC/WASH, Surveillance, and R&D. Impact-Effort analysis identified four perceived priority areas as “Quick Wins”: stewardship, IPC/WASH, surveillance, and animal health. No significant association was observed across sector (*P* = .91) or career stages (*P* = .13), indicating priorities are widely recognized across disciplines and career stages.

We acknowledge several limitations in this study. First, convenience sampling may have introduced selection bias toward stakeholders engaged in global AMR discourse. We mitigated this by recruiting across multiple sectors and experience levels. Second, free-text responses may introduce interpretative variability during coding. This was addressed using a structured thematic codebook and consensus review. Third, weighting (Top 1 = 3, Top 2 = 2, and Top 3 = 1) may overemphasize rank differences. We addressed this by reporting both weighted and unweighted results. Fourth, sparse cells in experience categories limited Chi-squared reliability; Fisher’s exact test was used instead. Fifth, although English only administration of the survey may have limited participation, we ensured the survey was distributed through multiple international AMR and One Health networks. Additionally, the professional sector categories used in this study were predefined by the research team, and respondents self-selected from nine fixed options. While this approach ensures consistency across responses, it does not capture differences within each broad category. For example, the clinical category may include professionals from different specialties such as infectious disease, surgery, or general practitioners, while the NGO category may include organizations with different roles and priorities. This may limit the interpretation of sector-specific findings and highlights the need for more detailed classifications in future studies. Finally, effort scores in the Impact-Effort analysis were based on informed authors consensus rather than formal Delphi procedures. We ensured scores were informed by existing literature applied using transparent criteria.

Notwithstanding these limitations, this study provides a stakeholder-led assessment of perceived gaps in AMR mitigation efforts using an open-ended question. Combining inductive coding with deductive alignment enhanced validity, while restructuring responses into long format enabled transparent quantification. The integration of weighted ranking and Impact-Effort analysis translated findings into actionable guidance.

The identification of antimicrobial stewardship and use as the most frequently cited perceived gap in AMR mitigation efforts (18.8% of responses, weighted score 119) presents a notable paradox. Despite being a cornerstone of the World Health Organization (WHO) Global Action Plan on AMR, and numerous national and professional guidelines,^4,17^ it surfaced as the most frequently cited perceived gap among stakeholders, pointing to a substantial disconnect between stewardship policy and how respondents experience its implementation on the ground.^18,19^ One respondent noted: *“Overuse and misuse of antimicrobials by unqualified persons, including quacks, and lack of supervision by trained professionals, especially in LMICs. Besides, there are no systems in place to check such activities.”* Another pointed to diagnostic limitation explaining that: *“In many regions, empirical antibiotic use is the default due to a lack of rapid diagnostics and microbiology capacity. This leads to broad-spectrum overuse, driving resistance.”*

Our findings suggest that respondents perceived this gap as stemming not from lack of technical feasibility but from insufficient political will and prioritization, inadequate funding, and a failure to adapt interventions to local contexts. These findings corroborate recent qualitative studies, which noted lack of clear political commitment, inadequate funding, and failure to adapt interventions to local context as major barriers to antimicrobial stewardship programs.^20^ As one respondent observed: *“Although AMR is recognized as a global threat by WHO and other UN agencies such as FAO, UNEP, and WOAH, their efforts to create integrated programs that reach all countries and people, especially in low-income countries and their indigenous peoples, are fragmented and inadequate.”* The widespread emphasis on stewardship across professional sectors and experience levels underscores global recognition of this issue. Addressing what respondents perceive as this gap requires shifting focus from creating more guidelines to ensuring their implementation.^20^ In our Impact-Effort Matrix analysis, antimicrobial stewardship and use was classified as a “Quick Win” (Impact score 119, effort 3/5, n = 58 mentions), suggesting that with the right political will and mobilized resources, this strategy can deliver high impact with relatively low to moderate effort.

Environmental AMR and waste was the second most cited perceived gap and the most frequent Top 1 priority ranking, reflecting a growing recognition of environmental transmission pathways. Once confined to specialist research communities, environmental AMR has become a mainstream concern among AMR stakeholders.^21^ This could be due to accumulating evidence that environmental reservoirs and transmission pathways substantially contribute to resistance dissemination.^22^ One respondent emphasized, *“I believe that focusing on antibiotic prescriptions is a mistake; we are overlooking a large part of the problem related to the environment and the plastisphere.”* Another noted, “*The abuse of antibiotics in farming; for example, most fish farmers tend to use antibiotics for their aquaculture and release these ‘antibiotics heavy laden’ wastewater without any form of pretreatment.”*

These perspectives highlight perceived gaps in environmental AMR mitigation efforts including wastewater disposal from healthcare facilities, pharmaceutical manufacturing, and livestock operations, which often contain high concentrations of antimicrobial residues, resistant bacteria strains, and mobile genetic elements encoding resistance.^23^ Classifying Environmental AMR and waste as a “Major Project” in our Impact-Effort analysis acknowledges the substantial infrastructure investments, regulatory frameworks, and multi-sectoral coordination required for effective intervention. International collaboration including support from the Quadripartite organizations (WHO, WOAH, FAO, UNEP) are essential as environmental AMR is inherently transboundary and no one country can address it alone.^5^

IPC/WASH, surveillance, and animal health were classified as “Quick Wins”, reflecting perceived implementation gaps by stakeholders despite strong evidence-base and inclusion in global frameworks.^4,24^ IPC/WASH received 9.4% of perceived gap mentions with a weighted score of 63 and was notably over-represented among second-tier priorities (16 mentions in the Top 2). This positioning suggests that IPC/WASH is seen by stakeholders as foundational yet complementary to more specialized interventions, a perception that may reflect persistent underestimation of its impact. Available studies and reports demonstrate that comprehensive IPC/WASH programs reduce healthcare-associated infections by 35%–70%, directly preventing the emergence and transmission of resistant bacteria. Every infection prevented eliminates the need for antimicrobial therapy, reducing selection pressure that drives resistance evolution.^24,25^ Despite this evidence, IPC implementation remains inadequate globally. A respondent from the Research/Academic sector said: *“Our work in low- and middle-income countries shows that while antibiotic use by people has a significant impact on carriage of resistant bacteria in hospitals, this isn’t the case in communities. Multiple studies reveal that community-level transmission factors, broadly associated with sanitation and hygiene, are closely associated with the probability of colonization with drug-resistant bacteria.”* Indeed, the WHO Global IPC Survey confirms that fewer than half of countries have established national IPC programmes with dedicated financing,^25^ and the emphasis on IPC by clinical professionals (15.0% of clinical priorities) reflects frontline recognition of these persistent gaps. Addressing what respondents identify as persistent IPC gaps requires sustained investment in healthcare workers’ training, and supervision, reliable water, sanitation, and hygiene infrastructure in healthcare facilities, and a supply chain system that ensures consistent availability of essential IPC supplies. The classification of IPC/WASH as a “Quick Win” confirms that high impact is achievable with relatively modest effort.

Awareness and education accounted for 8.4% of stakeholders perceived gap mentions, with a weighted score of 52, placing it near the boundary between Fill-Ins and Quick Wins. Evidence indicates that education yields impact primarily when combined with structural change, decision-support tools, and regulatory enforcement, rather than as a standalone measure.^26^ One respondent captured this tension: *“I can say we think we say it enough, but the impact is not there yet. People jump from one antibiotic to the other without thinking twice. A contributing factor is the fact that these antibiotics are readily available and accessible in Africa. I recently moved from Nigeria to Sweden, and I can tell the difference.”* The Fill-Ins classification of this theme reflects stakeholders’ recognition that education functions primarily as an enabler of other interventions.^27^ Its relatively low implementation cost makes it a valuable addition to comprehensive AMR programmes.

Surveillance, laboratory and data received 9.4% of perceived gap mentions (weighted score 59), reflecting stakeholders perceived challenges in AMR data generation, particularly in low- and middle-income countries (LMICs) where laboratory networks remain fragmented and under-resourced. Broad consensus across all sectors suggests current systems inadequately capture resistance patterns and trends. One respondent observed, *“Global AMR strategies still focus mainly on hospitals, yet most antibiotic use and transmission occur in the community.”* Although the WHO Global Antimicrobial Resistance and Use Surveillance System (GLASS) has expanded to over 100 countries, substantial gaps in data quality, representativeness, and geographic coverage persist.^14^ The “Quick Win” classification of this theme reflects opportunities to leverage existing infrastructure like tuberculosis and HIV laboratory networks in LMICs for targeted AMR capacity building. As one respondent recommended: *“Sewage surveillance of AMR is mandatory in EU now. It should become routine globally, so we can track the epidemic and global sharing of resistance gene alleles.”*

Animal health and food system received 8.1% of perceived gap mentions by stakeholders (weighted score: 54). This reflects stakeholders’ recognition that agricultural antimicrobial use, estimated to equal or exceed human use in many countries, drives substantial selection for resistance and environmental contamination. Although the use of antimicrobials as growth-promoting agents has been banned in many high-income countries, the practice continues in numerous LMICs where regulations are limited.^28^ The “Quick Win” classification of this theme reflects opportunities to engage agricultural sectors through established extension, improved practices, expanded vaccine access, and alternatives to prophylactic antimicrobial use. One respondent who identified cross-sectoral coordination as a central challenge said, *“Lack of effective communication and collaboration across sectors - despite awareness of the need for One Health approaches, it’s still challenging to implement these effectively; and to address the different incentives across sectors (economic pressures in agriculture, vs human health care and health care systems, etc.).*”

Three themes: access, equity and medicine quality (weighted score 41), financing, infrastructure and health systems (weighted score 21), and policy, governance and regulation (weighted score 17) were classified as “Hard Slogs” in our Impact-Effort analysis owing to their high implementation complexity relative to perceived impact. These represent enabling conditions rather than direct interventions, and their lower emphasis (access: 7.8% of responses) likely reflect stakeholders’ perception that they are broader health system challenges transcending AMR actions. Addressing medicine quality requires strengthened pharmaceutical regulation, supply chain integrity, and enforcement capacity. Similarly, improving equitable access requires health financing reforms, implementation of essential medicines lists, and supply chain strengthening that extend far beyond AMR. The minimal emphasis on financing and health system (3.9% of responses) likely reflects stakeholders’ perception of their status as foundational prerequisites for all health programs. Policy and governance received the second-lowest perceived gap mention (2.6% of responses), yet policy frameworks provide the essential foundation for nearly all AMR interventions. This minimal emphasis by stakeholders reveals potential blind spots, given that effective AMR interventions require policy environments providing legal authority, institutional mandates, and accountability mechanisms. Strategic approaches should embed policy advocacy within implementation, enabling pilot programmes to generate evidence for adoption and scale-up.^29^

One Health integration and coordination received the lowest perceived gap prioritization (2.3% of responses, weighted score 9) and, uniquely, zero Top 1 priority ranking, appearing exclusively in Top 2 and Top 3 rankings. This reveals a striking disconnect: while One Health dominates AMR policy discourse, implementation remains siloed and sector specific. Stakeholders seem to view coordination as instrumental infrastructure enabling concrete actions, rather than as a priority. This finding challenges how One Health is communicated and operationalized. Future AMR policies should emphasize concrete interventions with One Health frameworks rather than coordination for its own sake.

For policymakers and funders, the findings of these perceived gaps by AMR stakeholders suggest that resource allocation should emphasize scaling proven interventions (Quick Wins) while supporting long-term investment in complex challenges (Major Projects). Funding mechanisms must balance innovation with adequate support for foundational interventions such as stewardship, IPC, and surveillance as perceived by respondents as inadequately implemented in many settings despite their well-established status as priorities. For practitioners implementing AMR interventions, our findings highlight a recurring concern among respondents: evidence-based interventions developed in high-income settings require substantial contextual adaptation for successful implementation in resource-limited settings.^30^ Strengthening foundational interventions should precede or accompany efforts to address more complex challenges, and the environmental findings underscore the value of cross-sectoral collaboration to tackle multiple resistance drivers simultaneously.

Our study opens several avenues for future research. These include comparative analyses of perceived gaps against actual funding and policy attention, longitudinal tracking to determine whether priorities shift as context evolve, and intervention research testing whether addressing perceived gaps improves AMR outcomes.

### Conclusion

This study set out to examine stakeholder perceptions of gaps in AMR mitigation efforts across professional sectors and career stages, and to apply an Impact-Effort Matrix to support prioritization of One Health interventions. Across 309 priority statements from 103 professionals from 33 countries on 5 continents, antimicrobial stewardship and use emerged as the most frequently perceived gap, while environmental AMR and waste attracted the highest proportion of Top 1 priority ranking highlighting a widely shared concern that on-the-ground implementation has not kept pace with policy intent. The Impact-Effort Matrix identified four Quick Win areas that offer high impact with manageable implementation complexity, and three Hard Slogs requiring long-term structural investment. Stakeholder perceived gaps were consistent across professional sectors and career stages, suggesting these gaps reflect broad, cross-disciplinary concerns rather than sector-specific blind spots. The findings of this study provide actionable guidance and a decision-support lens for policymakers, funders, and implementers that may help in prioritizing One Health interventions.

## Supporting information

10.1017/ash.2026.10750.sm001 Ohemu et al. supplementary material Ohemu et al. supplementary material

## Supplementary material

The supplementary material for this article can be found at https://doi.org/10.1017/ash.2026.10750.

## Table 1. Long description

The table presents the distribution of 103 stakeholders across various sectors and their years of experience working in antimicrobial resistance (AMR). The sectors include Research/Academic, Clinical, Laboratory, Research & Development, Other, NGO, Government Agency, Program Management, and Policy. The Research/Academic sector has the highest representation with 44 respondents, accounting for 42.7 percent of the total, with a confidence interval of 33.2 to 52.6 percent. The Clinical sector follows with 20 respondents, or 19.4 percent, with a confidence interval of 12.4 to 28.4 percent. The Laboratory sector has 11 respondents, or 10.7 percent, with a confidence interval of 5.6 to 18.2 percent. Research & Development includes 8 respondents, or 7.8 percent, with a confidence interval of 3.5 to 14.7 percent. The Other sector has 7 respondents, or 6.8 percent, with a confidence interval of 2.9 to 13.5 percent. NGOs have 6 respondents, or 5.8 percent, with a confidence interval of 2.3 to 12.3 percent. Government Agencies have 5 respondents, or 4.9 percent, with a confidence interval of 1.7 to 11.0 percent. Program Management and Policy each have 1 respondent, or 1.0 percent, with confidence intervals of 0.0 to 5.3 percent. The years of experience in AMR are categorized into 0 to 4 years, 5 to 9 years, 10 to 19 years, and more than 20 years. The median years of experience is 7.0 years, with an interquartile range of 3.0 to 15.0 years. The 0 to 4 years category has 33 respondents, or 32.0 percent, with a confidence interval of 24.0 to 41.7 percent. The 5 to 9 years category has 26 respondents, or 25.2 percent, with a confidence interval of 17.4 to 34.6 percent. The 10 to 19 years category has 25 respondents, or 24.3 percent, with a confidence interval of 16.6 to 33.7 percent. The more than 20 years category has 19 respondents, or 18.4 percent, with a confidence interval of 11.6 to 27.2 percent.

Navigate back to Table 1..

## Table 2. Long description

A table categorizes themes related to antimicrobial resistance (AMR) mitigation efforts. Each theme includes definitions, inclusions, exclusions, and examples. Themes range from antimicrobial stewardship and environmental AMR to policy and governance, illustrating comprehensive strategies for addressing AMR.

Navigate back to Table 2..

## Table 3. Long description

The table presents data on perceived gaps/themes based on frequency of mention, weighted score, and priority rank from stakeholders’ responses. It includes 12 rows and 7 columns. The columns are labeled Rank, Perceived gap/Theme, n, percentage, Weighted Score, Top 1, Top 2, and Top 3. The rows list various themes such as Antimicrobial Stewardship and Use, Environmental AMR and Waste, Infection Prevention and Control, Surveillance, Laboratories and Data, R&D, Innovation and Diagnostics, Animal Health and Food Systems, Awareness, Education and Behaviour Change, Access, Equity and Medicine Quality, Financing, Infrastructure and Health Systems, Other / Cross-cutting, Policy, Governance and Regulation, and One Health Integration and Coordination. Notable trends include Antimicrobial Stewardship and Use having the highest weighted score of 119 and being ranked first by 21 stakeholders, while One Health Integration and Coordination has the lowest weighted score of 9 and is not ranked first by any stakeholder.

Navigate back to Table 3..

## Figure 1. Long description

A horizontal stacked bar chart compares the relative rank of various themes based on their priority levels, categorized into Top 1, Top 2, and Top 3. The x-axis represents the within-theme proportion in percentage, ranging from 0% to 100%. The y-axis lists the themes, including Antimicrobial Stewardship and Use, Environmental AMR and Waste, Surveillance, Laboratories and Data, Research and Development, Innovation and Diagnostics, Infection Prevention and Control, Awareness, Education and Behavior Change, Animal Health and Food Systems, Access, Equity and Medicine Quality, Financing, Infrastructure and Health Systems, Other Cross-cutting, Policy, Governance and Regulation, and One Health Integration and Coordination. Each bar is divided into three segments representing the priority ranks: light blue for Top 1, medium blue for Top 2, and green for Top 3. The chart illustrates how the relative importance assigned by respondents varies by theme, with proportions calculated using the total number of mentions per theme as the denominator. All values are approximated.

Navigate back to Figure 1..

## Figure 2. Long description

The bar graph compares the within-sector proportion of priority statements across different sectors related to antimicrobial resistance (AMR). The sectors include Clinical, Government Agency, Laboratory, Non-Governmental Organization (NGO), Other, Policy, Program Management, Research and Development, and Research/Academic. The graph is a stacked bar chart with horizontal bars. The x-axis represents the sectors, and the y-axis represents the within-sector proportion of priority statements in percentage. Each bar is divided into segments representing different themes, with a color scheme indicating various themes such as Access, Equity and Medicine Quality, Animal Health and Food Systems, Antimicrobial Stewardship and Use, Awareness, Education and Behavior Change, Environmental AMR and Waste, Financing, Infrastructure and Health Systems, Infection Prevention and Control (IPC/WASH), One Health Integration and Coordination, Other/Cross-cutting, Policy, Governance and Regulation, Research and Development, Innovation and Diagnostics, and Surveillance, Laboratories and Data. The graph shows the distribution of these themes within each sector, highlighting the varying priorities across different professional sectors. All values are approximated.

Navigate back to Figure 2..

## Figure 3. Long description

The bar graph compares the relative distribution of identified AMR priority themes across respondents with different years of professional experience, categorized into 0-4 years, 5-9 years, 10-19 years, and over 20 years. The graph is a stacked bar chart with horizontal bars. The x-axis represents the years of AMR experience categories, while the y-axis shows the within-experience proportion of priorities in percentage. Each bar is divided into segments representing different themes, with a color scheme indicating various themes such as Access, Equity and Medicine Quality, Animal Health and Food Systems, Antimicrobial Stewardship and Use, Awareness, Education and Behaviour Change, Environmental AMR and Waste, Financing, Infrastructure and Health Systems, Infection Prevention and Control (IPC/WASH), One Health Integration and Coordination, Other/Cross-cutting, Policy, Governance and Regulation, R&D, Innovation and Diagnostics, and Surveillance, Laboratories and Data. Early career professionals (0-4 years) emphasize Antimicrobial Stewardship and Use, Awareness, Education and Behaviour Change, and Environmental AMR and Waste. Mid-career stakeholders (5-9 years) prioritize Antimicrobial Stewardship and Use, Environmental AMR and Waste, and Infection Prevention and Control (IPC/WASH). Respondents with 10-19 years of experience focus on Environmental AMR and Waste, Antimicrobial Stewardship and Use, and Surveillance, Laboratories and Data. Senior professionals (20+ years) highlight Antimicrobial Stewardship and Use, Environmental AMR and Waste, and R&D, Innovation and Diagnostics. All values are approximated.

Navigate back to Figure 3..

## Figure 4. Long description

The heat map displays the distribution and intensity of AMR priority themes across various professional sectors. The map uses a grid layout with themes listed on the vertical axis and sectors on the horizontal axis. The color scale ranges from light blue to dark purple, indicating the count of priority statements, with darker shades representing higher counts. Notable concentrations of darker shading are observed in specific sector-theme combinations, highlighting areas of thematic emphasis. The themes include Surveillance, Laboratories & Data, R&D, Innovation & Diagnostics, Policy, Governance & Regulation, and others. Sectors range from Clinical to Research/Academic. The map reveals patterns of thematic concentration, with some sectors showing higher intensity in particular themes, suggesting areas of focused stakeholder concern and potential resource allocation.

Navigate back to Figure 4..

## Figure 5. Long description

The image is an Impact-Effort Matrix chart that visualizes the prioritization of AMR (Antimicrobial Resistance) themes based on their impact and implementation effort. The matrix is divided into four strategic quadrants: Quick Wins, Major Projects, Hard Slogs, and Fill-Ins. The x-axis represents the implementation effort, ranging from very easy to very difficult, while the y-axis represents the impact, measured by a weighted priority score. Each theme is plotted based on its impact and effort, with the size of the circles indicating the number of mentions. Themes in the Quick Wins quadrant, such as Antimicrobial Stewardship and Use, have high impact and low effort. Major Projects, like Environmental AMR and Waste, have high impact but require more effort. Hard Slogs, such as Financing, Infrastructure and Health Systems, have low impact and high effort. Fill-Ins, like One Health Integration and Coordination, have low impact and low effort. The chart also includes a legend explaining the strategic priority categories and the number of mentions.

Navigate back to Figure 5..
