# Supplementary material for: Stakeholder perceptions of gaps in antimicrobial resistance mitigation: a multinational multisectoral survey to inform prioritization of One Health interventions
Source: Antimicrob Steward Healthc Epidemiol. 2026 Jun 10;6(1):e174. doi: 10.1017/ash.2026.10750 (PMC13273150; doi:10.1017/ash.2026.10750)
Supplement: Ohemu et al. supplementary material [file S2732494X26107505sup001.docx]

**Supplementary Files**

**Stakeholder Perceptions of Gaps in Antimicrobial Resistance Mitigation: A Multinational Multisectoral Survey to Inform Prioritization of One Health Interventions.**

**Contents:**

S1. Distribution of perceived gaps by professional sector

S2. Distribution of perceived gaps by years of AMR experience

S3. Geographic distribution of respondents by country and continent

S4. Sector-specific dominant themes and illustrative respondent perspectives

S5. Continental distribution of AMR priority themes (descriptive)

**S1. Distribution of Perceived Gaps by Professional Sector**

Sector-specific differences were observed in perceived gaps in AMR mitigation strategies. Priority patterns were broadly consistent across sectors, with Antimicrobial Stewardship and Use consistently emphasized. No statistically significant association was observed between professional sector and priority theme (Fisher's exact test, p = 0.91).

**Table S1. Distribution of perceived gaps by professional sector, showing theme, frequency, total thematic mentions within sector, percentage, and 95% confidence interval.**

| **Sector** | **Perceived Gap / Theme** | **n** | **Total mentions within sector** | **%** | **95% CI** |
| --- | --- | --- | --- | --- | --- |
| Clinical | Access, Equity & Medicine Quality | 6 | 60 | 10.0 | 4.7–20.1 |
|  | Animal Health & Food Systems | 3 | 60 | 5.0 | 1.7–13.7 |
|  | Antimicrobial Stewardship & Use | 14 | 60 | 23.3 | 14.4–35.4 |
|  | Awareness, Education & Behavior Change | 5 | 60 | 8.3 | 3.6–18.1 |
|  | Environmental AMR & Waste | 7 | 60 | 11.7 | 5.8–22.2 |
|  | Financing, Infrastructure & Health Systems | 2 | 60 | 3.3 | 0.9–11.2 |
|  | Infection Prevention & Control (IPC/WASH) | 9 | 60 | 15.0 | 7.6–27.3 |
|  | One Health Integration & Coordination | 0 | 60 | 0.0 | – |
|  | Other / Cross-cutting | 3 | 60 | 5.0 | 1.7–13.7 |
|  | Policy, Governance & Regulation | 2 | 60 | 3.3 | 0.9–11.2 |
|  | R&D, Innovation & Diagnostics | 3 | 60 | 5.0 | 1.7–13.7 |
|  | Surveillance, Laboratories & Data | 6 | 60 | 10.0 | 4.7–20.1 |
| Government Agency | Access, Equity & Medicine Quality | 2 | 15 | 13.3 | 3.7–37.9 |
|  | Animal Health & Food Systems | 3 | 15 | 20.0 | 7.1–45.2 |
|  | Antimicrobial Stewardship & Use | 4 | 15 | 26.7 | 11.0–52.0 |
|  | Awareness, Education & Behavior Change | 1 | 15 | 6.7 | 1.2–29.8 |
|  | Environmental AMR & Waste | 2 | 15 | 13.3 | 3.7–37.9 |
|  | Financing, Infrastructure & Health Systems | 0 | 15 | 0.0 | – |
|  | Infection Prevention & Control (IPC/WASH) | 1 | 15 | 6.7 | 1.2–29.8 |
|  | One Health Integration & Coordination | 0 | 15 | 0.0 | – |
|  | Other / Cross-cutting | 0 | 15 | 0.0 | – |
|  | Policy, Governance & Regulation | 0 | 15 | 0.0 | – |
|  | R&D, Innovation & Diagnostics | 0 | 15 | 0.0 | – |
|  | Surveillance, Laboratories & Data | 2 | 15 | 13.3 | 3.7–37.9 |
| Laboratory | Access, Equity & Medicine Quality | 1 | 33 | 3.0 | 0.5–15.3 |
|  | Animal Health & Food Systems | 2 | 33 | 6.1 | 1.7–19.5 |
|  | Antimicrobial Stewardship & Use | 6 | 33 | 18.2 | 8.8–34.3 |
|  | Awareness, Education & Behavior Change | 3 | 33 | 9.1 | 3.2–23.6 |
|  | Environmental AMR & Waste | 5 | 33 | 15.2 | 7.0–30.8 |
|  | Financing, Infrastructure & Health Systems | 2 | 33 | 6.1 | 1.7–19.5 |
|  | Infection Prevention & Control (IPC/WASH) | 2 | 33 | 6.1 | 1.7–19.5 |
|  | One Health Integration & Coordination | 1 | 33 | 3.0 | 0.5–15.3 |
|  | Other / Cross-cutting | 1 | 33 | 3.0 | 0.5–15.3 |
|  | Policy, Governance & Regulation | 2 | 33 | 6.1 | 1.7–19.5 |
|  | R&D, Innovation & Diagnostics | 3 | 33 | 9.1 | 3.2–23.6 |
|  | Surveillance, Laboratories & Data | 5 | 33 | 15.2 | 7.0–30.8 |
| NGO | Access, Equity & Medicine Quality | 1 | 18 | 5.6 | 1.0–26.0 |
|  | Animal Health & Food Systems | 1 | 18 | 5.6 | 1.0–26.0 |
|  | Antimicrobial Stewardship & Use | 5 | 18 | 27.8 | 13.0–48.6 |
|  | Awareness, Education & Behavior Change | 2 | 18 | 11.1 | 3.1–33.1 |
|  | Environmental AMR & Waste | 1 | 18 | 5.6 | 1.0–26.0 |
|  | Financing, Infrastructure & Health Systems | 2 | 18 | 11.1 | 3.1–33.1 |
|  | Infection Prevention & Control (IPC/WASH) | 2 | 18 | 11.1 | 3.1–33.1 |
|  | One Health Integration & Coordination | 1 | 18 | 5.6 | 1.0–26.0 |
|  | Other / Cross-cutting | 2 | 18 | 11.1 | 3.1–33.1 |
|  | Policy, Governance & Regulation | 0 | 18 | 0.0 | – |
|  | R&D, Innovation & Diagnostics | 0 | 18 | 0.0 | – |
|  | Surveillance, Laboratories & Data | 1 | 18 | 5.6 | 1.0–26.0 |
| Other | Access, Equity & Medicine Quality | 2 | 21 | 9.5 | 2.6–28.9 |
|  | Animal Health & Food Systems | 3 | 21 | 14.3 | 5.0–34.6 |
|  | Antimicrobial Stewardship & Use | 3 | 21 | 14.3 | 5.0–34.6 |
|  | Awareness, Education & Behavior Change | 3 | 21 | 14.3 | 5.0–34.6 |
|  | Environmental AMR & Waste | 5 | 21 | 23.8 | 11.1–44.7 |
|  | Financing, Infrastructure & Health Systems | 0 | 21 | 0.0 | – |
|  | Infection Prevention & Control (IPC/WASH) | 2 | 21 | 9.5 | 2.6–28.9 |
|  | One Health Integration & Coordination | 0 | 21 | 0.0 | – |
|  | Other / Cross-cutting | 0 | 21 | 0.0 | – |
|  | Policy, Governance & Regulation | 1 | 21 | 4.8 | 0.9–22.7 |
|  | R&D, Innovation & Diagnostics | 1 | 21 | 4.8 | 0.9–22.7 |
|  | Surveillance, Laboratories & Data | 1 | 21 | 4.8 | 0.9–22.7 |
| Policy | Access, Equity & Medicine Quality | 0 | 3 | 0.0 | – |
|  | Animal Health & Food Systems | 0 | 3 | 0.0 | – |
|  | Antimicrobial Stewardship & Use | 1 | 3 | 33.3 | 6.1–79.2 |
|  | Awareness, Education & Behavior Change | 0 | 3 | 0.0 | – |
|  | Environmental AMR & Waste | 0 | 3 | 0.0 | – |
|  | Financing, Infrastructure & Health Systems | 0 | 3 | 0.0 | – |
|  | Infection Prevention & Control (IPC/WASH) | 0 | 3 | 0.0 | – |
|  | One Health Integration & Coordination | 0 | 3 | 0.0 | – |
|  | Other / Cross-cutting | 0 | 3 | 0.0 | – |
|  | Policy, Governance & Regulation | 0 | 3 | 0.0 | – |
|  | R&D, Innovation & Diagnostics | 1 | 3 | 33.3 | 6.1–79.2 |
|  | Surveillance, Laboratories & Data | 1 | 3 | 33.3 | 6.1–79.2 |
| Program Management | Access, Equity & Medicine Quality | 1 | 3 | 33.3 | 6.1–79.2 |
|  | Animal Health & Food Systems | 0 | 3 | 0.0 | – |
|  | Antimicrobial Stewardship & Use | 2 | 3 | 66.7 | 20.8–93.9 |
|  | Awareness, Education & Behaviour Change | 0 | 3 | 0.0 | – |
|  | Environmental AMR & Waste | 0 | 3 | 0.0 | – |
|  | Financing, Infrastructure & Health Systems | 0 | 3 | 0.0 | – |
|  | Infection Prevention & Control (IPC/WASH) | 0 | 3 | 0.0 | – |
|  | One Health Integration & Coordination | 0 | 3 | 0.0 | – |
|  | Other / Cross-cutting | 0 | 3 | 0.0 | – |
|  | Policy, Governance & Regulation | 0 | 3 | 0.0 | – |
|  | R&D, Innovation & Diagnostics | 0 | 3 | 0.0 | – |
|  | Surveillance, Laboratories & Data | 0 | 3 | 0.0 | – |
| Research & Development | Access, Equity & Medicine Quality | 1 | 24 | 4.2 | 0.7–20.2 |
|  | Animal Health & Food Systems | 2 | 24 | 8.3 | 2.3–25.9 |
|  | Antimicrobial Stewardship & Use | 1 | 24 | 4.2 | 0.7–20.2 |
|  | Awareness, Education & Behavior Change | 2 | 24 | 8.3 | 2.3–25.9 |
|  | Environmental AMR & Waste | 5 | 24 | 20.8 | 9.6–40.4 |
|  | Financing, Infrastructure & Health Systems | 1 | 24 | 4.2 | 0.7–20.2 |
|  | Infection Prevention & Control (IPC/WASH) | 3 | 24 | 12.5 | 4.4–30.4 |
|  | One Health Integration & Coordination | 0 | 24 | 0.0 | – |
|  | Other / Cross-cutting | 1 | 24 | 4.2 | 0.7–20.2 |
|  | Policy, Governance & Regulation | 0 | 24 | 0.0 | – |
|  | R&D, Innovation & Diagnostics | 7 | 24 | 29.2 | 15.2–48.6 |
|  | Surveillance, Laboratories & Data | 1 | 24 | 4.2 | 0.7–20.2 |
| Research/Academic | Access, Equity & Medicine Quality | 10 | 132 | 7.6 | 4.1–13.4 |
|  | Animal Health & Food Systems | 11 | 132 | 8.3 | 4.8–13.9 |
|  | Antimicrobial Stewardship & Use | 22 | 132 | 16.7 | 11.1–24.3 |
|  | Awareness, Education & Behavior Change | 10 | 132 | 7.6 | 4.1–13.4 |
|  | Environmental AMR & Waste | 26 | 132 | 19.7 | 14.1–26.9 |
|  | Financing, Infrastructure & Health Systems | 5 | 132 | 3.8 | 1.6–8.6 |
|  | Infection Prevention & Control (IPC/WASH) | 10 | 132 | 7.6 | 4.1–13.4 |
|  | One Health Integration & Coordination | 5 | 132 | 3.8 | 1.6–8.6 |
|  | Other / Cross-cutting | 4 | 132 | 3.0 | 1.2–7.5 |
|  | Policy, Governance & Regulation | 3 | 132 | 2.3 | 0.8–6.5 |
|  | R&D, Innovation & Diagnostics | 14 | 132 | 10.6 | 6.4–17.0 |
|  | Surveillance, Laboratories & Data | 12 | 132 | 9.1 | 5.3–15.2 |

*Values represent the frequency of thematic mentions within each professional sector. Percentages reflect within-sector proportions. 95% confidence intervals calculated using the Wilson score method. Rows with 0 mentions are included for completeness. Sectors with n = 1 (Policy, Program Management) are presented for transparency but were excluded from statistical comparison due to sparse cell counts.*

**S2. Distribution of Perceived Gaps by Years of AMR Experience**

Priority patterns were broadly consistent across experience categories, with Antimicrobial Stewardship and Environmental AMR emphasized across career stages. No statistically significant association was observed between years of AMR experience and priority theme selection (Fisher's exact test with Monte Carlo simulation, 10,000 replicates, p = 0.13).

**Table S2. Distribution of perceived gaps by years of professional AMR experience, showing experience category, theme, frequency, total mentions within category, percentage, and 95% confidence interval.**

| **Experience category** | **Perceived Gap / Theme** | **n** | **Total mentions within category** | **%** | **95% CI** |
| --- | --- | --- | --- | --- | --- |
| 0–4 years | Access, Equity & Medicine Quality | 9 | 99 | 9.1 | 4.8–16.5 |
|  | Animal Health & Food Systems | 6 | 99 | 6.1 | 2.8–12.7 |
|  | Antimicrobial Stewardship & Use | 16 | 99 | 16.2 | 10.4–24.4 |
|  | Awareness, Education & Behavior Change | 14 | 99 | 14.1 | 8.8–21.8 |
|  | Environmental AMR & Waste | 14 | 99 | 14.1 | 8.8–21.8 |
|  | Financing, Infrastructure & Health Systems | 8 | 99 | 8.1 | 4.1–15.3 |
|  | Infection Prevention & Control (IPC/WASH) | 10 | 99 | 10.1 | 5.6–17.4 |
|  | One Health Integration & Coordination | 3 | 99 | 3.0 | 1.0–8.5 |
|  | Other / Cross-cutting | 6 | 99 | 6.1 | 2.8–12.7 |
|  | Policy, Governance & Regulation | 1 | 99 | 1.0 | 0.2–5.5 |
|  | R&D, Innovation & Diagnostics | 5 | 99 | 5.1 | 2.2–11.3 |
|  | Surveillance, Laboratories & Data | 7 | 99 | 7.1 | 3.4–14.0 |
| 5–9 years | Access, Equity & Medicine Quality | 5 | 78 | 6.4 | 2.8–14.1 |
|  | Animal Health & Food Systems | 8 | 78 | 10.3 | 5.3–18.9 |
|  | Antimicrobial Stewardship & Use | 18 | 78 | 23.1 | 15.1–33.6 |
|  | Awareness, Education & Behavior Change | 6 | 78 | 7.7 | 3.6–15.8 |
|  | Environmental AMR & Waste | 11 | 78 | 14.1 | 8.1–23.5 |
|  | Financing, Infrastructure & Health Systems | 2 | 78 | 2.6 | 0.7–8.9 |
|  | Infection Prevention & Control (IPC/WASH) | 10 | 78 | 12.8 | 7.1–22.0 |
|  | One Health Integration & Coordination | 1 | 78 | 1.3 | 0.2–6.9 |
|  | Other / Cross-cutting | 4 | 78 | 5.1 | 2.0–12.5 |
|  | Policy, Governance & Regulation | 2 | 78 | 2.6 | 0.7–8.9 |
|  | R&D, Innovation & Diagnostics | 6 | 78 | 7.7 | 3.6–15.8 |
|  | Surveillance, Laboratories & Data | 5 | 78 | 6.4 | 2.8–14.1 |
| 10–19 years | Access, Equity & Medicine Quality | 5 | 75 | 6.7 | 2.9–14.9 |
|  | Animal Health & Food Systems | 7 | 75 | 9.3 | 4.6–17.9 |
|  | Antimicrobial Stewardship & Use | 14 | 75 | 18.7 | 11.4–29.1 |
|  | Awareness, Education & Behavior Change | 4 | 75 | 5.3 | 2.1–12.8 |
|  | Environmental AMR & Waste | 17 | 75 | 22.7 | 14.1–34.3 |
|  | Financing, Infrastructure & Health Systems | 1 | 75 | 1.3 | 0.2–7.0 |
|  | Infection Prevention & Control (IPC/WASH) | 2 | 75 | 2.7 | 0.7–9.1 |
|  | One Health Integration & Coordination | 2 | 75 | 2.7 | 0.7–9.1 |
|  | Other / Cross-cutting | 0 | 75 | 0.0 | – |
|  | Policy, Governance & Regulation | 3 | 75 | 4.0 | 1.4–11.2 |
|  | R&D, Innovation & Diagnostics | 9 | 75 | 12.0 | 6.2–21.7 |
|  | Surveillance, Laboratories & Data | 11 | 75 | 14.7 | 8.4–24.6 |
| 20+ years | Access, Equity & Medicine Quality | 5 | 57 | 8.8 | 3.9–18.8 |
|  | Animal Health & Food Systems | 4 | 57 | 7.0 | 2.7–17.2 |
|  | Antimicrobial Stewardship & Use | 10 | 57 | 17.5 | 9.9–29.1 |
|  | Awareness, Education & Behavior Change | 2 | 57 | 3.5 | 1.0–11.9 |
|  | Environmental AMR & Waste | 9 | 57 | 15.8 | 8.5–27.4 |
|  | Financing, Infrastructure & Health Systems | 1 | 57 | 1.8 | 0.3–9.3 |
|  | Infection Prevention & Control (IPC/WASH) | 7 | 57 | 12.3 | 6.0–23.2 |
|  | One Health Integration & Coordination | 1 | 57 | 1.8 | 0.3–9.3 |
|  | Other / Cross-cutting | 1 | 57 | 1.8 | 0.3–9.3 |
|  | Policy, Governance & Regulation | 2 | 57 | 3.5 | 1.0–11.9 |
|  | R&D, Innovation & Diagnostics | 9 | 57 | 15.8 | 8.5–27.4 |
|  | Surveillance, Laboratories & Data | 6 | 57 | 10.5 | 4.9–21.1 |

*Values represent the number and proportion of priority statements attributed to each theme within experience categories. 95% confidence intervals calculated using the Wilson score method. Rows with 0 mentions are included for completeness.*

**S3. Geographic Distribution of Respondents by Country and Continent**

Respondents represented 33 countries across five continents. The highest participation came from Nigeria (n=17) and Kenya (n=14), followed by South Africa (n=8), the United Kingdom (n=8), and India (n=6). Six respondents reported working at a global or regional level and were not assigned to a specific country. Country of work was provided as a free-text response. Continent was self-selected from a predefined list; no assignments or reclassifications were made by the research team.

**Table S3a. Distribution of respondents by self-reported country of work (n = 103).**

| **Country of Work** | **Continent (self-reported)** | **n** |
| --- | --- | --- |
| Nigeria | Africa | 17 |
| Kenya | Africa | 14 |
| South Africa | Africa | 8 |
| United Kingdom | Europe | 8 |
| India | Asia | 6 |
| United States | North America | 6 |
| France | Europe | 3 |
| Singapore | Asia | 3 |
| Uganda | Africa | 3 |
| Bangladesh | Asia | 2 |
| Cameroon | Africa | 2 |
| Canada | North America | 2 |
| Gambia | Africa | 2 |
| Ghana | Africa | 2 |
| Australia | Asia | 1 |
| Brazil | South America | 1 |
| Denmark | Europe | 1 |
| Ethiopia | Africa | 1 |
| Finland | Europe | 1 |
| Ireland | Europe | 1 |
| Italy | Europe | 1 |
| Malawi | Africa | 1 |
| Mexico | North America | 1 |
| Mongolia | Asia | 1 |
| Nepal | Asia | 1 |
| Pakistan | Asia | 1 |
| Spain | Europe | 1 |
| Switzerland | Europe | 1 |
| Tanzania | Africa | 1 |
| Timor-Leste | Asia | 1 |
| Tunisia | Africa | 1 |
| Turkey | Asia | 1 |
| Zambia | Africa | 1 |
| Global scope* | – | 6 |
| **Total** |  | **103** |

**Six respondents reported working at a global or regional level and were not assigned to a specific country. Country of work was provided as a free-text response. Continent was self-selected from a predefined list of six options (Africa, Asia, Europe, North America, South America, and Oceania); no assignments or reclassifications were made by the research team. One respondent selected Asia despite being based in Australia, which was retained as given. Oceania was included as a response option but was not selected by any respondent.*

**Table S3b. Distribution of respondents by self-selected continent of work (n = 103).**

| **Continent (self-reported)** | **n** | **%** |
| --- | --- | --- |
| Africa | 54 | 52.4 |
| Europe | 20 | 19.4 |
| Asia | 18 | 17.5 |
| North America | 10 | 9.7 |
| South America | 1 | 1.0 |
| **Total** | **103** | **100.0** |

*Continent of work was self-selected from a predefined list. Percentages may not total 100 due to rounding.*

**S4. Sector-Specific Dominant Themes and Illustrative Respondent Perspectives**

For each sector, dominant priority themes were identified based on weighted thematic analysis and top-ranked priorities. Descriptive summaries capture typical sectoral emphases, and illustrative verbatim quotations provide contextual insights into how priorities manifest across practice, policy, research, and implementation settings. Quotes were selected to represent the range of perspectives within each theme, prioritizing statements that articulated the theme clearly or captured a nuance not apparent from the quantitative data alone.

**Table S4. Sector-specific contrast in perceived gaps in AMR mitigation, dominant thematic emphases, and illustrative respondent perspectives.**

| **Sector** | **Dominant themes** | **Typical emphasis** | **Illustrative respondent perspectives** |
| --- | --- | --- | --- |
| **Clinical** | Antimicrobial Stewardship & Use; Infection Prevention & Control (IPC/WASH); Environmental AMR & Waste; Surveillance, Laboratories & Data | Stewardship implementation; diagnostics and surveillance gaps; over the counter sales/prescribing in LMICs | *“Overuse and misuse of antimicrobials by unqualified persons, including quacks, and lack of supervision by trained professionals, especially in LMICs. Besides, there are no systems in place to check such activities”* |
| **Government Agency** | Antimicrobial Stewardship & Use; Animal Health & Food Systems; Environmental AMR & Waste | Regulation and enforcement gaps; animal health prioritization; surveillance infrastructure | *“limited regulation and enforcement on access and use of antimicrobials in animals. These antimicrobials end up being overused and misused enabling an incubation pathway for resistant bugs that later end up in humans or ecosystems.”* |
| **Laboratory** | Antimicrobial Stewardship & Use; Environmental AMR & Waste; Surveillance, Laboratories & Data | Environmental transmission pathways; behavioral and economic barriers; governance gaps in LMICs; Access to affordable antimicrobials | *“A lack of integrated surveillance and data infrastructure is a significant challenge. The WHO calls for integrated systems across human, animal, and environmental sectors, yet many remain siloed. In regions like Kenya, inadequate routine surveillance and insufficient data hinder tracking resistance trends and evaluating interventions. Robust, timely data is essential for early detection and preventing the spread of AMR.”* |
| **NGO** | Antimicrobial Stewardship & Use; Awareness, Education & Behavior Change; Other / Cross-cutting | Community-level stewardship; Behavior change; Inadequate investment in surveillance; Waste management | *"Proper waste management of unused or expired antimicrobials in the community(households).& Detection of these pharmaceutical waste residues in the environment as part of one health surveillance."* |
| **Other** | Environmental AMR & Waste; Animal Health & Food Systems; Awareness, Education & Behavior Change | Agricultural practice; silo working; cross-sector communication failures; environmental pathways | *"* *Everyone is getting in the space of AMR control with little or no knowledge of what it really is. I feel there should be some kind of prior knowledge test before we certify public health professionals to be trained on basics of AMR and capable of leading AMR programs. I recommend national bodies as well as academic bodies to lead AMR efforts and all agencies interested in working in the AMR space should tie up with such bodies. It would also throw a lens on types of stakeholders in this space and efforts being conducted, that can be further harmonized through a dedicated group of advisors."* |
| **Policy** | Antimicrobial Stewardship & Use; Surveillance, Laboratories & Data; R&D, Innovation & Diagnostics | Diagnostic utilization; data quality and surveillance; diagnostic solutions | *“Unwillingness for governments to pay for solutions, including investing in incentives for new antibiotics and reimbursement for diagnostic solutions.”* |
| **Research & Development** | R&D, Innovation & Diagnostics; Environmental AMR & Waste; Infection Prevention & Control (IPC/WASH) | Pharmaceutical waste disposal; airborne AMR risks; environmental exposure routes; IPC/WASH | *"The development of new and effective antibiotics for vulnerable populations, including children, and especially newborn babies who are very susceptible to infections."* |
| **Research/Academic** | Environmental AMR & Waste; Antimicrobial Stewardship & Use; R&D, Innovation & Diagnostics | Long-term environmental effects; antibiotic residues in wastewater; food animal pathways; vaccination | *"Transmission of AMR and resistance genes through transportation of people and animals.”* |
| **Program Management** | Antimicrobial Stewardship & Use; Access, Equity & Medicine Quality | Medicine quality, affordability, and treatment adherence | *"Adherence to treatment with side effects."* |

*Dominant priority themes were identified based on weighted thematic analysis. Illustrative quotes are presented verbatim from respondents' free-text responses. Sectors with n = 1 (Policy, Program Management) are included for completeness.*

**S5. Continental Distribution of AMR Priority Themes (Descriptive)**

The table below presents the distribution of AMR priority themes across self-reported continents of work. These data are presented for descriptive purposes only. Given the unequal representation across continents, particularly the very small sample sizes for North America (n=10) and South America (n=1), no statistical comparisons across continents were performed. Africa (n=54) and Europe (n=20) have the largest representation and provide the most stable descriptive estimates. Readers should interpret regional patterns in that light.

**Table S5. Continental distribution of AMR priority themes based on self-reported continent of work, showing frequency and within-continent percentage for each theme across Africa, Asia, Europe, North America, and South America.**

| **Theme** | **Africa** | **Asia** | **Europe** | **North America** | **South America** | **Total** |
| --- | --- | --- | --- | --- | --- | --- |
| Antimicrobial Stewardship & Use | 36 (22.2%) | 8 (14.8%) | 11 (18.3%) | 2 (6.7%) | 1 (33.3%) | 58 |
| Environmental AMR & Waste | 22 (13.6%) | 10 (18.5%) | 11 (18.3%) | 7 (23.3%) | 1 (33.3%) | 51 |
| R&D, Innovation & Diagnostics | 5 (3.1%) | 9 (16.7%) | 8 (13.3%) | 7 (23.3%) | 0 | 29 |
| Infection Prevention & Control (IPC/WASH) | 17 (10.5%) | 5 (9.3%) | 4 (6.7%) | 3 (10.0%) | 0 | 29 |
| Surveillance, Laboratories & Data | 15 (9.3%) | 2 (3.7%) | 10 (16.7%) | 2 (6.7%) | 0 | 29 |
| Awareness, Education & Behavior Change | 19 (11.7%) | 3 (5.6%) | 3 (5.0%) | 1 (3.3%) | 0 | 26 |
| Animal Health & Food Systems | 12 (7.4%) | 3 (5.6%) | 4 (6.7%) | 6 (20.0%) | 0 | 25 |
| Access, Equity & Medicine Quality | 15 (9.3%) | 4 (7.4%) | 5 (8.3%) | 0 | 0 | 24 |
| Financing, Infrastructure & Health Systems | 8 (4.9%) | 2 (3.7%) | 0 | 1 (3.3%) | 1 (33.3%) | 12 |
| Other / Cross-cutting | 8 (4.9%) | 1 (1.9%) | 1 (1.7%) | 1 (3.3%) | 0 | 11 |
| Policy, Governance & Regulation | 3 (1.9%) | 3 (5.6%) | 2 (3.3%) | 0 | 0 | 8 |
| One Health Integration & Coordination | 2 (1.2%) | 4 (7.4%) | 1 (1.7%) | 0 | 0 | 7 |

*Values are reported as n (%), where n represents the number of priority statements assigned to each theme within a continent. Percentages reflect within-continent emphasis rather than contributions to the global total. Continent of work was self-selected by respondents. No statistical comparisons across continents were performed due to unequal and insufficient representation in some regions, particularly North America (n=10) and South America (n=1). These data are presented for descriptive purposes only.*
